# In Hot Water: Global Warming Takes a Toll on Coral Reefs

**DOI:** 10.1289/ehp.116-a292

**Published:** 2008-07

**Authors:** Charles W. Schmidt

In the summer of 2005, while Atlantic hurricanes battered coastlines from Cuba to Mexico, the Eastern Caribbean baked under a relentless sun with barely a breeze to cool the air. Tourists and locals alike wilted in the heat, and below the sea, marine life and corals in particular suffered as well. The windless calm settled in just as a buildup of unusually warm water began accumulating in the region. Ordinarily, easterly trade winds would have churned the sea, helping it to cool. But thanks to an unprecedented heat wave beginning in May—the result of a confluence of factors related to climate change, scientists say—water temperatures in the Eastern Caribbean climbed and stayed high for months, reaching levels that by September would be warmer than any recorded in 150 years.

The heat disturbed a symbiotic partnership that coral animals normally maintain with a type of algae called zooxanthellae. Zooxanthellae supply corals with essential nutrients produced by photosynthesis, particularly carbon, in return for the shelter and access to sunlight provided by the reefs. The algae also impart color to the corals, which themselves are colorless. But as sea temperatures rose, the zooxanthellae disappeared, leaving their carbon-deprived hosts behind to starve. The reefs turned snow white, the color of the underlying stonelike structures they had built up over centuries, in a phenomenon known as coral bleaching.

As the heat wave progressed, it left a trail of bleached reefs the likes of which had never been seen in the Caribbean. By year’s end, coral cover ranging from 90% in the Virgin Islands to 52% in the French West Indies was affected.

Coral bleaching isn’t always fatal—if water temperatures cool in time, the zooxanthellae might return, allowing corals to recover. But in parts of the Eastern Caribbean, the reefs never got a chance. Almost as soon as their recovery started, they were attacked by diseases affecting a range of coral species down to 60 feet. By 2007, roughly 60% of the coral cover in the Virgin Islands and 53% in Puerto Rico’s La Parguera Natural Reserve was dead—an unprecedented tragedy.

The Eastern Caribbean disease outbreak came on the heels of what’s been a rough several decades for coral reefs worldwide. Long suffering from land-based pollution, habitat destruction, and overfishing, coral reefs now must also contend with climate change, which has accelerated their global decline. This puts a wealth of biodiversity at risk. Reefs support up to 800 types of coral, 4,000 fish species, and countless invertebrates. Reef-dwelling species numbering in the hundreds of thousands may not even be catalogued yet, some scientists speculate.

The implications of these declines could be as disastrous for human health as they are for marine life. Globally, reefs provide a quarter of the annual fish catch and food for about 1 billion people, according to the United Nations Environment Programme. Reefs protect shorelines from storm surges, which could become more powerful as sea levels rise with climate change. Tourism—a mainstay of coastal economies in the tropics, worth billions in annual revenue—could suffer if reefs lose their appeal.

Reefs are also a long-standing source of medicines to treat human disease. Being attached to reefs, corals and other immobilized marine animals can’t escape predators, so they deploy a range of chemical compounds to deter hunters, fight disease, and thwart competing organisms. Two antiviral drugs (vidarabine and azidothymidine) and the anticancer agent cytarabine were developed using compounds extracted from Caribbean reef sponges. Another product called dolastatin 10, isolated from the sea hare (*Dolabella auricularia*) of the Indian Ocean, has been investigated as a treatment for breast and liver cancers and leukemia. Many more lifesaving medicines and useful chemical products could one day be derived from reef dwellers, experts say.

Saving these ecosystems is imperative on a range of levels, says Caroline Rogers, a marine ecologist with the U.S. Geological Survey in St. John, U.S. Virgin Islands. “We have to save them for economic, ecological, aesthetic, and even spiritual reasons,” she says. “People need to feel connected with nature and with systems that are bigger than they are. Coral reefs are awe-inspiring—we’re losing something that we barely understand.”

## Threats from Climate Change

Corals live within a few degrees of temperatures that can send them into a tailspin. They’ve survived on that precipice for thousands of years because ocean temperatures in the tropics have been stable. But that’s no longer the case. Seawater warmed by a global average of nearly 1°C over the twentieth century, according to the United Nations Intergovernmental Panel on Climate Change (IPCC). Meanwhile, episodes of coral bleaching and disease are occurring with mounting frequency around the world. In 1997–1998, the world’s largest bleaching event ever killed 16% of the world’s reefs, with mortality approaching 90% throughout Bahrain, the Maldives, Sri Lanka, Singapore, and parts of Tanzania.

If carbon dioxide in the atmosphere rises from its current level of 380 ppm to 450–500 ppm by mid-century, as the IPCC predicts could happen if greenhouse gas emissions are not curbed, average ocean temperatures will rise an additional 2°C, an intolerable increase for most coral species. What’s more, atmospheric carbon dioxide is being absorbed by seawater and converted to carbonic acid, which serves to lower the ocean’s pH, threatening reef structures with dissolution, explains Ove Hoegh-Guldberg, director of the Centre for Marine Studies at the University of Queensland in Australia. Scientists now warn that within a few decades, reefs could suffer cataclysmic changes, as coral populations dwindle past the point of return.

Rogers and colleague Jeff Miller, a fisheries biologist with the National Park Service in St. John, witnessed the Virgin Islands reef crash that began in 2005. Miller’s job requires him to perform annual surveys on reef sites throughout the Virgin Islands National Park, which extends outward from St. John into offshore areas. But when sea temperatures spiked from the high 20s to over 30°C, leading some corals to bleach, Miller quadrupled his monitoring efforts to see how the reefs were faring. Submerged in the crystal blue water, he and his colleagues watched as the bleaching outbreak mushroomed far beyond their expectations. Then, just as the corals began to recover, diseases took hold, delivering a lethal blow. Erinn Muller, then a researcher with the U.S. Geological Survey who was working with Rogers, quantified the mortality from disease, counting more than 6,000 disease patches (or “lesions”).

If Miller and Muller hadn’t beefed up the monitoring schedule, the link between coral bleaching, disease, and death in the Eastern Caribbean wouldn’t have been made. “The newly exposed skeleton is rapidly overgrown by ‘turf algae,’ and then you can’t be sure what caused the mortality,” Miller explains. “If we hadn’t expanded our monitoring, we might have assumed bleaching killed the reefs, when that wasn’t the case. We used to see disease as a source of chronic, low-level mortality. This outbreak showed [diseases] can be catastrophic and devastating over short durations.”

Miller and Muller’s work sends the urgent message that diseases can destroy reefs at rates far greater than once assumed, says Matt Patterson, the South Florida/Caribbean network coordinator with the National Park Service in Palmetto Bay, Florida. What’s more, he adds, it shows how little scientists know about the effects of warming on coral health.

The association between bleaching and warming is fairly well established, but scientists still can’t agree on how rising temperatures produce this effect. Hoegh-Guldberg says warming may trigger the release of zooxanthellae-killing toxins by *Vibrio* bacteria, whereas others suggest heat-stressed corals give off acidic free radicals that drive the algae away. Still others believe both scenarios are plausible. Meanwhile, links between warming and disease have only recently gained traction among scientists, although that picture too is complicated; diseases don’t always follow bleaching, and sometimes they occur in the absence of warmer water.

The fact that coral diseases in the Eastern Caribbean erupted in the aftermath of a thermal anomaly bolsters hypotheses that the two are somehow related, says Drew Harvell, a professor of ecology and evolutionary biology at Cornell University and chair of the Coral Reef Targeted Research Program’s Coral Disease Working Group. “Associations between warming and infectious disease in corals are getting more established now,” she says. “That Eastern Caribbean outbreak was massive, the biggest to occur in the region that we know of. My concern is that it’s also a harbinger of things to come.”

## Pinpointing Pathogens

Like other veterinary conditions, coral diseases are caused by infectious agents such as bacteria, viruses, and fungi. But the field of coral pathology is in its infancy, with perhaps only five of what could be dozens of diseases linked to specific microbes so far. The most common affliction during the Eastern Caribbean outbreak was white plague, so named because the disease kills tissue and exposes the coral’s ivory-colored skeleton. Purportedly caused by the bacterium *Aurantimonas coralicida*, white plague accounted for 98% of coral disease during the outbreak, affecting colonies of brain corals (*Diploria* spp.) and the more common boulder star corals (*Montastraea annularis*). Another disease known as white pox increased in severity on elkhorn corals (*Acropora palmata*). Now listed as threatened under the Endangered Species Act, this coral once formed massive interlocking colonies throughout the Caribbean. (White pox has been linked to *Serratia marcescens*, a ubiquitous bacterium from the human gut, among dozens of other sources. Some scientists speculate the illness can be set off by sewage contamination, but so far there’s been no field evidence to support that hypothesis.)

Scientists speculate that warming triggers coral illness by stressing the animals, leaving them open to infection. That’s particularly true if diseases follow bleaching, which deprives corals of carbon, leaving them dependent on whatever passing prey they can grab with their tentacles. “The corals are exhausted, so they get sick,” explains Patterson. “It’s like what would happen to you if you worked seventy-hour work weeks without a good diet and a break.”

In humans, stress invites illness by wearing down immunity, and evidence suggests this may also hold true for corals. In unpublished research, Kim Ritchie, a microbiologist with the Mote Marine Laboratory in Sarasota, Florida, recently found that warmer temperatures can invoke a potentially lethal shift in the corals’ microbial makeup. Corals are normally sheathed in a slimy biofilm loaded with beneficial antibiotic-producing (“probiotic”) bacteria that fight off other pathogenic strains. But when water temperatures rise, the probiotic bacteria disappear. “You simply can’t find them,” Ritchie says. In their absence, she adds, reef pathogens may move in and wreak havoc.

Working in her laboratory, Ritchie is trying to find out which bacteria keep pathogens in check, and how. Those investigations could do much to reveal fundamental aspects of coral immunity. But they also hold out a promise for treatment: in aquarium studies Ritchie and colleagues are investigating the feasibility of using probiotic bacteria to keep diseases at bay. Ritchie notes that probiotics are indigenous to coral ecosystems. What’s more, she says, probiotic bacteria already have a safe history in aquaculture, where they are used to control pathogens in farmed Atlantic salmon.

Meanwhile, Christina Kellogg, an environmental microbiologist with the U.S. Geological Survey in St. Petersburg, Florida, and Eugene Rosenberg, a professor of molecular microbiology and biotechnology at Tel Aviv University in Israel, are independently investigating a different treatment approach that relies on bacteriophages (“phages” for short), which unlike broad-spectrum antibiotics usually kill only bacteria of a single, targeted strain. Long used by Russian and Balkan doctors for human medicine, phage therapy fell out of favor in the West when antibiotics came on the scene in the 1950s. Rosenberg claims to have found two varieties that kill coral pathogens, including the bacterial cause of a white plague–like disease. Based on these findings, he concludes that coral diseases might one day be limited by phage treatment.

Harvell applauds this work as “very good, exciting, pioneering work,” although she notes “management potential would, however, be far down the road.” Ritchie, however, points to one drawback of phage therapy: “There is surely not one nor even a few pathogens causing problems, but many types of bacteria that take advantage of stressed corals and aid in the disease process,” she says. “[Phages] are specific to a particular bacterial strain, so researchers will have to isolate and culture a phage for every potential coral pathogen, which isn’t feasible.” On the other hand, she adds, research into both phage therapy and the use of beneficial bacteria to control pathogens could lead to important advances in the understanding of coral health.

Still, a number of prominent Australian researchers dispute the feasibility and safety of treating coral disease medically on such a large scale. “History is blighted with biological and ecological interventions that have failed dismally at best; more often they have been disastrous failures,” says Paul Marshall, director of the Climate Change Group with the Great Barrier Reef Marine Park Authority in Townsville, Australia. “Meddling with physiological processes at ecosystem scales will introduce more risk than it alleviates.”

Hoegh-Guldberg believes biological interventions won’t work because—in his view—coral diseases aren’t even caused by primary pathogens, as Rosenberg and others suggest they are. Instead, he proposes, coral diseases are merely secondary infections triggered when reactions caused by heat stress turn normally harmless bacteria into opportunistic killers.

Moreover, Hoegh-Guldberg maintains that even if medical treatments were identified, using them wouldn’t be practical given the scale of the resource at risk. He points out that coral reefs worldwide are estimated to cover nearly 300,000 square kilometers; the prospect of treating this large area is, he says, mind-boggling. “Simply put,” he concludes, “there are very few avenues for responding to the crisis that coral reefs face. The main thing we have to do is massively reduce emissions of greenhouse gases into the atmosphere. This is about as urgent as it gets.”

## The Immediate Challenge

While few would dispute that limiting greenhouse gases is good for corals, Miller says the more immediate goal should be to block the known stressors that kill them daily: pollutants, sewage contamination, and sediments from unpaved roads and land development. Throughout the tropics, development cuts deep ravines into coastal hillsides, generating flows of earth that tumble to the sea. Floating “silt curtains” can capture some of that material before it reaches the ocean, but these devices are rarely used and can be installed incorrectly. As a result, sediments settle over the reefs, with a range of consequences. Bleached reefs, weakened from zooxanthellae loss, are particularly vulnerable to the stress of the increased sediment load, says C. Mark Eakin, Coral Reef Watch coordinator for the National Oceanic and Atmospheric Administration (NOAA). “[The corals are] spending the limited energy they have on cleaning out sediments instead of capturing food,” he explains. Sediments pose another problem, Eakin adds, in that they coat the skeletal reef surfaces that coral larvae would otherwise settle on and populate. In the corals’ place, weedy turf algae can move in and further impede healthy reef growth and development.

Reef management strategies espoused by NOAA and other agencies strive in part to limit human contacts during peak thermal stress, which could help corals conserve the energy they need to overcome bleaching and other heat-related health problems, asserts Heidi Schuttenberg, a PhD candidate at James Cook University in Queensland, Australia. “You want to help bleached corals survive while they’re starving,” she explains. “Sediments [stirred up by] dredging or fin-kicks are especially harmful to bleached reefs. And bleached corals have to go through a period of recovery, which occurs through spawning and larval recruitment, which are some of the most vulnerable states in a coral’s life cycle.”

Along these lines, protections should be directed especially toward resilient reefs with the greatest potential to thrive in the long run, Marshall and Schuttenberg suggest. As to what makes reefs resilient, Marshall and Schuttenberg both say the ability to survive bleaching is a determining factor. Other crucial factors relate to what experts call connectivity, which involves access to deep, cool currents that carry an abundance of coral larvae from “source reefs.”

Billy Causey, the Southeast regional director of NOAA’s National Marine Sanctuary Program in Key West, Florida, encourages dive shops, dredge operators (who remove sediments to create channels in the sea), and other businesses to avoid resilient reefs when bleaching risk is high. The response so far has been cooperative, he remarks. But if conditions warrant, Causey adds he can also restrict public access to the reefs by law, given that the entire Florida Keys National Marine Sanctuary—which he oversees—has been designated a “marine protected area” (MPA) by NOAA and the State of Florida. “We don’t like to affect businesses,” he says, “but if they go out there when corals are threatened, that could lead to problems.”

Around the world, MPAs are among the main instruments governments use to protect threatened ocean resources. Camilo Mora, a postdoctoral fellow at the Scripps Institution of Oceanography in San Diego, California, whose investigations in this area were published 23 June 2006 in *Science*, says nearly 20% of the world’s coral reefs fall within MPAs, which offer protections that vary by country and by regions within countries. Still, MPA enforcement tends to be lax and management insufficient, Mora claims, especially with respect to limits on fishing.

Fish play a crucial role in reef health. Herbivorous species such as parrotfish, for instance, graze on the weedy turf algae that invade reef surfaces, thus preparing those surfaces for larval recruitment. Now, with more gastronomically desirable reef species such as grouper in decline from overfishing, parrotfish have become highly sought after. And that’s ironic, Miller adds, given that parrotfish were once viewed as trash species to be avoided. In the 1 November 2007 issue of *Nature*, Peter Mumby, a professor of marine ecology at Exeter University in the United Kingdom, published an article in which he called for urgent action to protect parrotfish in order to prevent coral reefs from becoming overgrown with algae and seaweed.

But in the United States, MPAs generally don’t limit fishing at all, leaving that task to the more stringent and less abundant “marine reserves,” where resource extraction is banned. Establishing MPAs and marine reserves is politically challenging, owing to backlash from affected stakeholders including business owners, says Causey. For instance, it took several years and the involvement of nearly two dozen organizations to create the Florida Keys MPA, he says.

Both Miller and Rogers now wonder if threats posed by climate change—being that they emerge from broader forces beyond local control—will undermine political support for MPAs and marine reserves. “The 2005–2006 [coral bleaching] event here was so ubiquitous it ignored political designations like country, territory, or MPA,” Miller says. “It had a massive footprint on the whole region. So our concern is that these warming-related losses might influence perceptions of whether MPAs are successful at doing what they’re supposed to do, which is to protect the resource. If you’re a politician, it might be hard to argue that MPAs can weather these types of events.”

From the front lines of reef decline, Miller and Rogers have also seen how local businesses—even as they lament coral loss—seem unwilling to accept the cost of slowing it. The researchers say it’s easier for businesses to point at climate change or even coral toxicity from trace residues of suntan lotion (as described in the April 2008 issue of *EHP*)—issues they can’t do anything about—than it is for them to accept financial responsibility for paving roads, limiting sewage and septic releases, or undertaking other, more burdensome measures to protect coral reefs. Meanwhile, the researchers admit, it’s all too easy to ignore the reefs’ plight, being that these treasured resources lie under the sea surface, out of sight, and maybe for too many people, also out of mind.

## Figures and Tables

**Figure:**
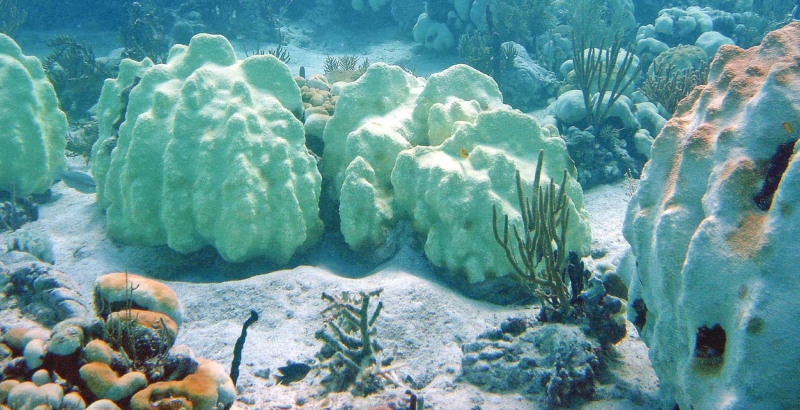


**Figure:**
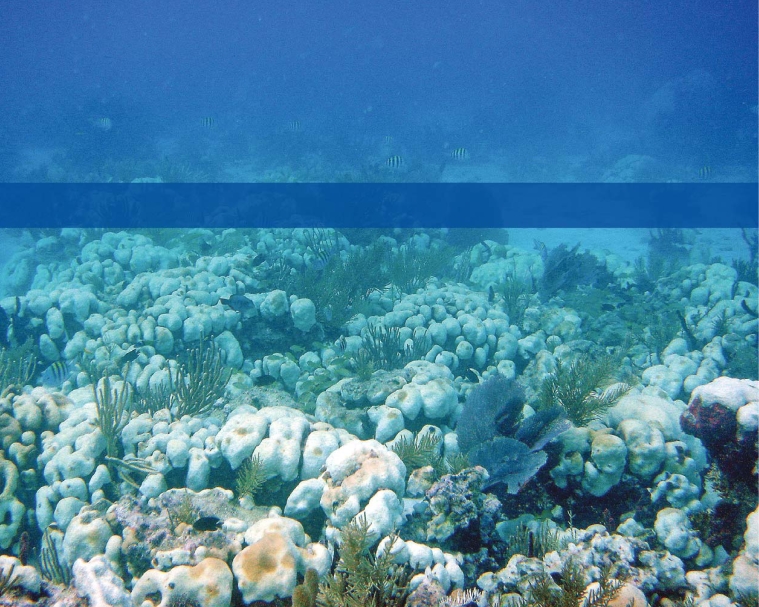
Coral bleaching, Virgin Islands National Park This 2005 bleaching event was the prelude to unprecedented coral deaths in the Eastern Caribbean.

**Figure:**
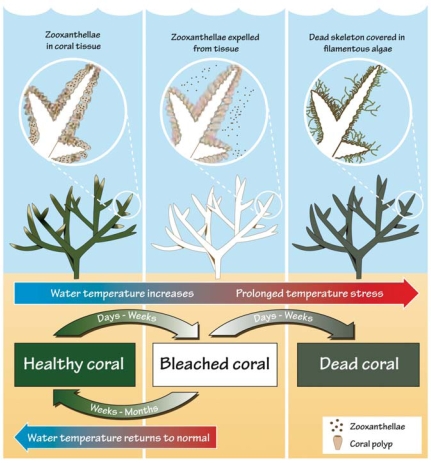
Stages of coral stress If water temperatures increase above a critical threshold, typically over a large area, corals will begin to lose their zooxanthallae. The underlying white structure becomes visible. At this stage, the bleached corals can still regain their zooxanthallae if stressful conditions subside soon enough. However, should temperature stress continue, the corals are likely to die. **Source:** Marshall P, Schuttenberg H. 2006. A reef manager's guide to coral bleaching. Townsville, Australia: Great Barrier Reef Marine Park Authority; p. 7.

**Figure:**
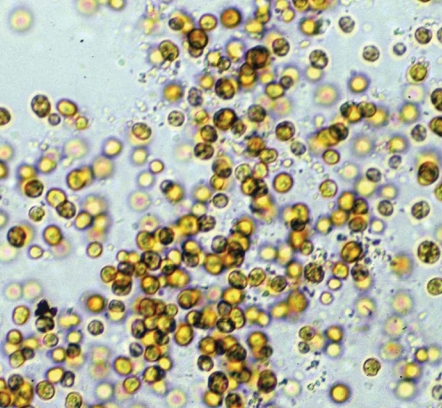
Perfect partner This light microscopy photo shows *Symbiodinium* zooxanthellae cultured from rice coral. The tiny zooxanthellae live within most types of coral polyps. They give the coral its color but more importantly help the coral survive by providing it with food produced through photosynthesis. In turn, the coral polyps provide the zooxanthellae with a protected environment and the nutrients they need to carry out photosynthesis.

**Figure:**
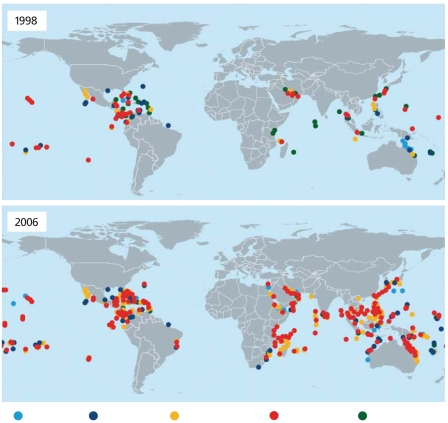
Global trends in coral bleaching The extent and severity of coral bleaching events have increased over the last decade. Prior to 1998 coral bleaching had been recorded in most of the world’s main reef regions, but few reefs had experienced severe bleaching. Since 1998, however, every reef region has experienced severe bleaching, with many areas suffering significant bleaching-induced mortality. **Source:** Marshall P, Schuttenberg H. 2006. A reef manager's guide to coral bleaching. Townsville, Australia: Great Barrier Reef Marine Park Authority; p. 5.

**Figure:**
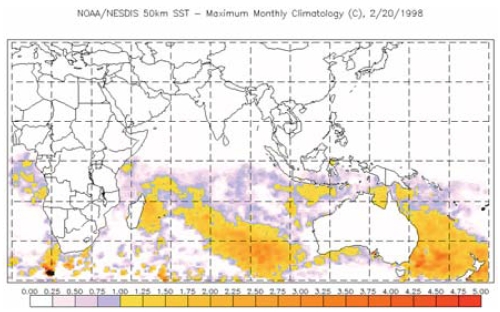
NOAA HotSpot map for the Eastern Hemisphere, 20 February 1998 Elevated water temperatures in 1998 spurred the worst bleaching event to date. According to the Union of Concerned Scientists, bleaching was reported in 60 countries around the world, and Indian Ocean corals were hit the worst. Temperature anomalies of 1–2ºC extending over a period of days to weeks should alert reef managers that a medium to high risk of bleaching exists. **Source:** NOAA

**Figure:**
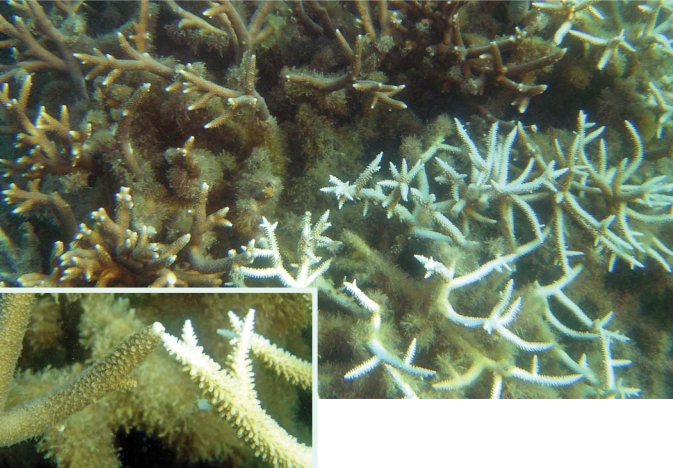
Acroporid corals undergoing bleaching, Guam, October 2006 Bleached coral can recover if larval coral (“recruits”) find their way to the bare branches. However, weedy “turf algae” may take over the bleached coral first, making it impossible for recruits to repopulate the reef.

**Figure:**
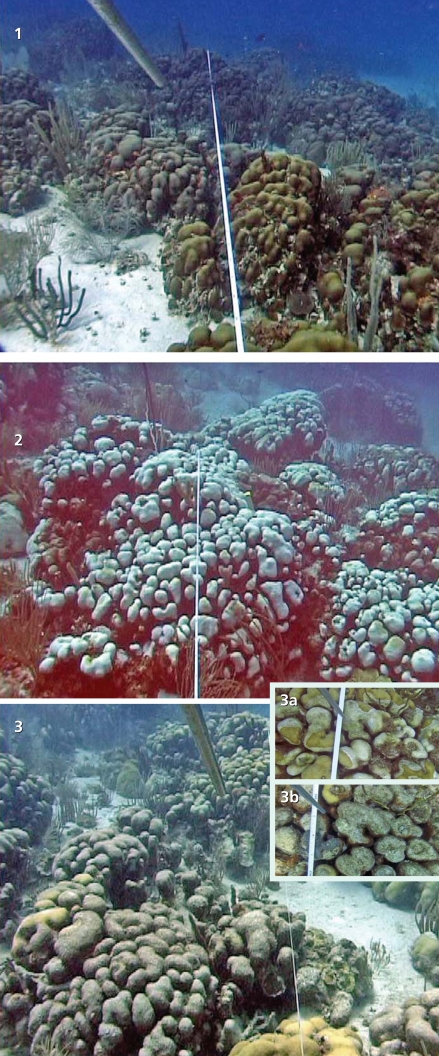
Loss progression among boulder star corals, Buck Island Reef National Monument, U.S. Virgin Islands (1) Healthy corals before bleaching; (2) corals undergoing bleaching; (3) corals that have succumbed to disease after bleaching. Insets show the same patch of coral during disease (3a) and after death (3b).

**Figure:**
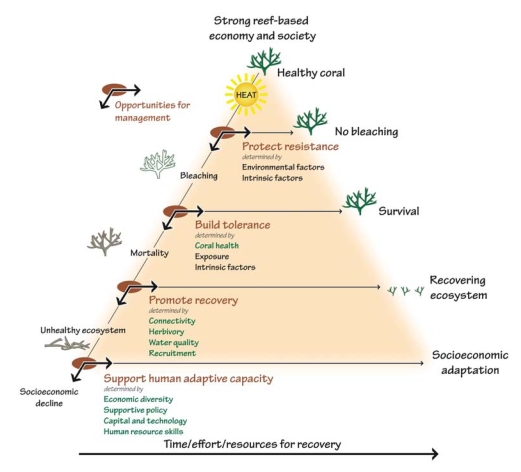
Opportunities for reef management intervention Four conditions determine how well reefs weather thermal stress: bleaching resistance, coral tolerance, reef recovery, and human adaptive capacity. Each condition is influenced by a suite of factors, some of which can be influenced by local management actions (shown above in green) and some of which cannot (shown above in black). **Source:** Marshall P, Schuttenberg H. 2006. A reef manager's guide to coral bleaching. Townsville, Australia: Great Barrier Reef Marine Park Authority; p. 10.

